# Does the cognitive therapy of depression rest on a mistake?

**DOI:** 10.1192/pb.bp.115.052936

**Published:** 2017-10

**Authors:** Richard G. T. Gipps

**Affiliations:** 1 Oxford University, UK

## Abstract

Cognitive therapy for depression is common practice in today's National Health Service, yet it does not work well. Aaron Beck developed it after becoming disillusioned with the psychoanalytic theory and therapy he espoused and practised. But Beck's understanding of psychoanalysis appears to have been seriously flawed. Understood rightly, the psychoanalytic approach offers a cogent theory and therapy for depression which, unlike the cognitive approach, takes us to its emotional-motivational roots. A clinically successful therapy can afford to eschew theory and rest on its pragmatic laurels. This is not the case for cognitive therapy. The time is right to re-examine the psychoanalytic theory and treatment of depression.

Cognitive–behavioural therapy (CBT) is, alongside antidepressant medication and counselling, today's mainstay treatment for depression in the UK. Such treatments tend to work better than nothing at all,^1^ yet in general fare little better than placebo,^2,3^ suffer relatively high relapse rates, and often struggle to provide a complete remission.^4,5^

CBT treatments for anxiety disorders, by comparison, appear more successful.^6^ They work by helping the patient articulate, then transcend, their underlying inchoate fears, the transformative learning happening directly within action and experience. This bottom-up experiential focus contrasts with the cognitive treatment of depression, where the patient is more typically trained in an arduous top-down task of managing unhealthy habits of mind through attentional and behavioural training and rational self-management.^7^

One reason for the rather low remission and high relapse rates for CBT-treated depression may be that the treatment does not reach to the emotional roots of the problem. That CBT principally theorises and treats the maintaining, rather than identifies underlying, causes of depression was acknowledged by Aaron Beck, the American psychiatrist who developed the cognitive aspect of this pragmatic depression treatment in the 1950s.^7^

The psychoanalytic psychology Beck displaced had a theory of the root cause of depression: avoidance of intolerable emotion blocks healthy emotional functioning, depletes uncontrived self-possession, and lowers self-esteem.^8^ But his clinical experience (as a psychoanalytic psychotherapist) and scientific research (on depression, anger and dreaming) led Beck to discount the significance of such unconscious emotion. Ever the pragmatist, he focused instead on treating the conscious assumptions and ruminations of the patient which, he proposed, were maintaining their depressive state.

This article argues that the versions of psychoanalytic theory and therapy Beck espoused, practised and then rejected were recondite and implausible. The claim is that Beck mistakenly threw out the psychoanalytic baby of a significant psychological understanding and treatment of depression with the bathwater of a rather idiosyncratic understanding of psychoanalytic theory and practice. The thesis offered is theoretical rather than scientific: it considers the fundamental conceptual matter of how that theory is itself to be understood, rather than the secondary empirical matter of evidence for or against hypotheses derived from particular interpretations of it. But given the availability of far more plausible versions of psychoanalytic theory and practice that outline and treat depression at its emotional roots, and given the relatively poor success of CBT for depression, the time is now right to put the psychoanalytic theory back on the table and test hypotheses and therapies derived from it.

## Psychopathological theory

An important theme in psychoanalytic psychopathology has depression resulting from the avoidance of feelings of loss. When the acute sadness of letting go of a beloved or an aspiration is too painful, the patient may avoid it by shutting down and instead become flat and morose. This, however, prevents adaptation to loss, since sadness is simply the most fundamental form that our recognition of loss takes. Another especially significant theme has depression resulting from the unconscious avoidance of anger towards those to whom we are attached. Rather than risk the feared relational fallout from expressing anger, the patient unconsciously depletes herself, trading that sense of self-worth that would be provided by an angrily assertive sense of injustice for the stability of her relationship. A third theme has depression resulting from the unconscious avoidance of fear. Rather than face the vicissitudes of uncontrollable fate in one's love and work, the patient instead constructs and lives in a dismal and diminished version of himself, his situation and his future. Even if life thereby becomes grim, at least now it will not take him by surprise.

Such ideas form but three strands of a more complex psychoanalytic conceptualisation of depression,^8–10^ but are fairly widespread in both popular and clinical culture for good reason. For clinically what we discover, again and again in straightforward cases, is a depressed person who avoids sadness, fear and in particular anger by going flat, downgrading her sense of her own value, shutting down self-assertion, not allowing herself to get even reasonable hopes up, falsely characterising herself as perpetrator rather than victim of relational injustices, characterising herself as deserving of treatment which reasonable others would consider unjust, denying the significance of her unmet emotional needs, and envisaging a world in which the exercise of agency appears foolhardy. In more complex cases, however, we find masochistic self-abasement added to an anger-avoiding dynamic: unconscious hatred towards another breaks through the attempts at self-suppression and gives rise to intolerable guilt, and this in turn inspires self-punishment where the anger towards the other is ‘retroflected’ (taken out on oneself), leading to further and darker melancholic misery.^9^

It was by way of a reaction against such psychoanalytic theory that a young, psychoanalytically minded American psychiatrist and dream researcher developed the theory and practice of cognitive therapy. Following a helpful personal experience of psychoanalysis, Aaron Beck treated several depressed patients using psychoanalytical methods, applied (albeit unsuccessfully) for membership of the American Institute of Psychoanalysis, and published a few papers on psychoanalytic psychotherapy and on the themes of his depressed patients' dreams.^11,12^ Reading these today we learn in particular of his scientific interest in the increased prevalence of thwarted, deprived, excluded, rejected, injured and ashamed themes in his patients' dreams, and of his clinical interpretation of these along psychoanalytic lines.

Already in these early papers, however, we find a curious feature which presages his later rejection of psychoanalysis: although most of the themes Beck describes (e.g. ‘I was in a restaurant but the waiters would not serve me’; ‘Everyone was invited to the party but me’; ‘My fiancée married someone else’) appear interpretable in terms of the simple hypothesis of motivated self-depletion, surprisingly he interprets them all in terms of the more complex dynamic of self-hatred: the depressed patient's misery is always seen as deliberately rather than incidentally self-inflicted, reflecting his ‘need to suffer’.^13^ Dreams such as not getting food that is requested, or being rejected – which in themselves appear at most to indicate a need to safely anticipate setbacks or protect cherished others from one's resentment by portraying life as hopeless and oneself as worthless – are instead counterintuitively read as ‘the representation of self-punitive tendencies … the depressed person feels guilt about his ego-alien drives and punishes himself for them.’^11^

The question naturally arises as to why Beck was so drawn to the masochism hypothesis. And this is particularly significant because it was when his later experimental and clinical investigations – including his patients' appropriately negative reactions to interpretations overly organised by this hypothesis – rightly led him to doubt whether he really was meeting everywhere with self-hatred, that he threw out the entirety of the baby of the general psychoanalytic theory of depression along with the specific counterintuitive bathwater of an over-reaching application of the masochism hypothesis. Which is to say that the entire project of explaining why a patient may be unconsciously motivated to think and feel and act in depressive ways was abandoned; in Beck's hands their condition now collapsed into a habitual rut of self-maintaining negative thought, feeling and behaviour.

One answer to why Beck was so compelled by the masochism hypothesis that it overrode his recognition of the frequent sufficiency of the simpler theory of motivated self-depletion is apparent in the early papers themselves. Beck somehow entirely forgets about the psychoanalytic ideas of depression as due either to thwarted mourning or to avoided fear, becoming solely preoccupied by the idea of it as due to suppressed anger. Such depression as did not appear to evince anger (since, one imagines, it was really due to suppressed sadness or fear) could then only be brought into line with the suppressed anger hypothesis by positing that such a patient was masochistically contriving to make herself depressed.

Another answer only becomes apparent in Beck's later writings; it concerns the nature of dreaming. (Grasping this takes a little patience, but it is worth the effort.) In these later writings he tells us that what he was actually trying to do in his early research was to set the clinical psychoanalytic theory of depression as a function of suppressed anger on a firmer scientific basis by providing quantitative psychological evidence of unconscious anger in the dreams of his depressed patients.^14,15^ Although he did not report it at the time, what he later tells us he found is what has also been established since:^16^ that as a group people with depression have fewer themes of anger in their dreams than people who do not have depression. This puzzled him, as somehow he had understood the Freudian idea of dreams being the ‘royal road to the unconscious’^17^ to mean that feelings unacceptable to the waking patient ought to show up straightforwardly in their dreams.^14^ The finding of fewer angry themes in the dreams of patients with depression therefore appeared to contradict the psychoanalytic hypothesis of depression as resulting from suppressed anger. This troubled Beck, but – at least until he found independent evidence of the implausibility of this interpretation – he realised he could save the psychoanalytic theory by interpreting the very dreaming of such miserabilist dreams as masochistically motivated (‘he makes himself dream such miserable dreams because he hates himself’).

What is deeply unclear in all of this is why Beck thought that Freud's theory posited that unconscious emotions ought to be directly manifest and countable in dreams. After all, Freud's theory was that dreams serve to protect sleep by helping prevent the dreamer's anxious recognition of emotions they find unacceptable, such as anger towards loved ones. In that theory dream construction involves the disguise – through displacement, condensation, reversal, negation and projection – of such impulses and emotions as threaten a comfortable sense of self–other relations. (Freud's ‘royal road’ refers not to a direct, undisguised revelation of the unconscious, but rather to dreams offering particularly rich sites for interpreting the products of defences against intolerable feelings and motivations – by contrast with the myriad, emotionally irrelevant concerns of waking life.) In retrospect it seems at least possible that Beck's enthusiasm to formulate and test a psychoanalytic hypothesis using the quantitative methods of empirical psychology ended up getting the better of his grasp of the psychoanalytic theory itself.

To sum up so far: a central plank of cognitive therapy's origin myth has it that it developed out of an apparent scientific disconfirmation of the clinical psychoanalytic theory of depression as a motivationally explicable state.^14^ But in retrospect what seems more likely to have happened is that an inappropriate quantitative methodology deployed to provide support for an unlikely theory of depressive dreaming actually found against it; that an implausibly general theory of depressive masochism was developed to save the floundering analytic theory; and that when this overly general masochism theory was dropped for good reason, the whole idea of symptoms as motivated by the avoidance of intolerable feelings – i.e. the whole idea of a depth psychology – was jettisoned for no good reason at all.

## Therapeutic practice

As described above, the development of cognitive therapy's psychopathological theory rests on its unwarranted rejection of the psychoanalytic notion of depression as unconsciously motivated. The development of cognitive therapy's psychotherapeutic technique, however, depends on its rejection of the centrality for psychotherapeutic practice of what psychoanalysis terms the ‘transference relationship’.^7^ A curious aspect, then, of Beck's development of cognitive therapy is that it was actually inspired by his encounter with, and dawning realisation of the clinical significance of, what are clearly recognisable as his patients' transferences to him.

First, a note on ‘transference’. A defining preoccupation of psychoanalysis is with how immersion in relationships which inspire concern and attachment – such as those with psychotherapists, partners, parents, employers, etc. – so readily elicits unrealistic fearful and idealising expectations concerning others' views of us. These relentlessly maintained, emotionally charged expectations are seen by psychoanalysis as being at the root of much psychopathology, and their manifestations inside and outside of therapy are known as negative and positive transferences. They can be easy to attend from, as it were, but powerfully difficult to attend to – i.e. they are often unconscious – and their patterns are typically transferred from one significant other to another over time.^18,19^ The task of psychoanalytic therapy is the patient's emancipation from distorting transference patterns, a task prosecuted by first facilitating the flourishing and then the subsequent experiential emancipation from the transferences between patient and psychotherapist, an experience that can then generalise to the transferences in the rest of the patient's life. Much of a psychoanalytical psychotherapist's training has to do with developing his ability to make room for and be emotionally touched, yet not inexorably swept along, by the patient's transference so he can think about, experience, describe, and help liberate the patient from her unconscious depressogenic emotional habits.

To return to cognitive therapy. As Beck^15^ tells the story, he had a patient who would lie on the couch each session and regale him with lurid tales of her sexual exploits, while he sat back and offered somewhat by-the-book psychoanalytic interpretations regarding the content of whatever it had occurred to her to say. At the end of one session, however, Beck asked his patient how she was feeling; she replied ‘anxious’. Beck first suggested to her, in an interpretation focusing only on intra-psychic issues, that conflicts about sex were making her anxious. She cautiously responded, however, to the effect that her real worry was interpersonal: her worry was that he was bored by her. Beck then began to see ‘that there's a whole stream of pre-conscious thinking that goes on that the patient doesn't normally communicate to the analyst’^15^ – especially pessimistic, biased, black-and-white, over-general irrational expectations concerning what the therapist thinks and feels about the patient. Beck came to call these transference expectations the patient's ‘negative automatic thoughts’ (NATs) and, drawing on the ‘rational therapy’ of Albert Ellis,^20^ went on to develop a significant range of procedures to help the patient attend to and challenge their NATs.

There are several curious things about this and related narratives Beck offers.^21^ The first is that Beck the novice psychoanalyst started out (as one does) by naively listening to and interpreting the explicit content of what the patient freely said, rather than listening in with an analytic ear to what she was not saying, to the unconscious dimension of the transference (i.e. listening to how he featured latently in her mind), or listening in to his own countertransference (i.e. to the feelings provoked in him by, in particular, the performative rather than declarative aspect of her discourse). Thus, despite the patient's manifest lack of inhibition in talking about sex, Beck still interprets her anxiety as due to sexual conflict.

The second is that when Beck shows a real interest in his patient, asking her how she feels, she is able to acknowledge her transference to him, and they can understand it together to beneficial effect. We go on to hear that these worries (which, despite, or perhaps because of, being so very omnipresent in her mind she never discussed before) are actually common for her in other settings too. As the therapeutic relationship is strengthened (by Beck's concerned question about her actual feelings), the emotionally alive experience of the transference (her worries about Beck being bored by her) also begins to be acknowledged and worked through, and interpretative speculation about intra-psychic conflict is foregone.

The most striking thing about Beck's narrative, however, is that this therapy-potentiating emotional experience of the transference is set aside almost as soon as it is encountered. Anyone who has been in psychotherapy will know how replete it is with holding back acknowledgement, both to oneself and to the therapist, of one's thoughts and impulses for fear of encountering one's own or the therapist's disapproval, despite such fears speaking right to the heart of such emotional difficulties as brought one to therapy in the first place. Notwithstanding the simplicity of the ‘fundamental rule’ of psychoanalysis – to ‘free associate’, i.e. say whatever is actually on your mind (which is not the same as saying whatever you feel like saying!) – the fact is that no one can truly follow it,^21,22^ since we naturally associate away from rather than towards conflictual emotional preoccupations.^23^ This is why the therapist's job is often to listen not so much to the content of what is said as to performative matters of style, timing and omission. Beck, however, construes NATs as merely incidentally hard for the patient to articulate and challenge – due to a lack of training in attending to and reporting on them^21^ – rather than because of their emotional valence. This, I submit, is intuitively implausible. More consonant with everyday clinical and personal experience is the notion that his patient did not elaborate her actual worries because she feared they might not be disconfirmed – and chose instead to distract herself and please him with endless talk about sex, presumably since, as we all know, Freudians do have rather a reputation for being interested in such matters!

Cognitive therapists are often accused of ignoring the importance of the therapeutic relationship, but as Beck's daughter Judith Beck explains, this is false – cognitive therapy ‘requires a good therapeutic relationship. Therapists do many things to build a strong alliance. For example, they work collaboratively with clients … ask for feedback… and conduct themselves as genuine, warm, empathic, interested, caring human beings.’^24^ However, as psychoanalytical psychotherapist Jonathan Shedler responds, ‘This is the kind of relationship I would expect from my hair stylist or real estate broker. From a psychotherapist, I expect something else. [Beck appears] to have no concept that the therapy relationship provides a special window into the patient's inner world, or a relationship laboratory and sanctuary in which lifelong patterns can be recognized and understood, and new ones created.’^25^ Shedler's optimism regarding his hair stylist and estate agent perhaps warrants some cognitive restructuring, but his point about the therapeutic relationship stands.

A relationship which is not merely instrumentally useful (as intended by cognitive therapy), but itself intended as the unique locus of change (as in psychoanalytic therapy), is one which both activates the patient's latent transference fears (that the therapist is untrustworthy, angry etc.) and simultaneously provides enough of a working alliance to enable such prototypical fears to be experienced, understood and worked through in real time. With a merely collaborative and empathic focus the opportunity is lost for the real-time eliciting and challenging of the patient's underlying emotional preoccupations. The result is somewhat like trying to conduct exposure therapy for a phobia without physically encountering the fearful stimulus, or like a chat between two adults about the difficulties of a child left waiting in the next room.

A popular canard has it that psychoanalytic psychotherapy is unhelpfully preoccupied with the past, whereas CBT is practically focused on the present. This ignores the way both therapies tend to formulate current disturbance in terms of childhood-acquired pathogenic beliefs. More importantly, it ignores the fact that, at the level of technique, CBT tends to focus on matters arising in the patient's past week, whereas a transference-focused psychotherapist hones in on uncomfortable transference feelings alive right now between patient and therapist. Rather than providing merely intellectual insight to further an ongoing project of dreary self-management, itself supplementing an already exhausting project of defensively managing intolerable feelings, psychoanalytic psychotherapy instead offers an intrinsically mutative emotional exchange which already constitutes a growth in self-possession and a change of heart, obviating the need for such self-management.

## Implications

Work in the transference is designed to facilitate a patient's living exposure to their real underlying fears about how they would be seen if they were to allow themselves their own true feelings. The opportunity is thereby provided for a true change of heart – i.e. for emancipation from depressive cognition through an experiential recovery, acceptance and integration of hitherto unconscious emotional experience. Ideally, this would reduce the need to manage the dismal distal products of this emotional evasion with therapeutic techniques of behavioural activation, cognitive challenge or mindfulness. The result of such an effective therapy for depression would be akin to that sometimes achieved by CBT for anxiety conditions: a transformative learning, from the experiential ground up, that reinstates true self-possession.

The time is past for studies comparing outcomes of self-professed cognitive therapy/CBT and psychoanalytic practitioners. The apparent success of particular therapies in such trials is better predicted and explained not by therapeutic model^26^ but by the theoretical orientation of the lead experimenter,^27^ the personal qualities of the therapists,^28^ or by theory drawn from quite different models.^29^ Beck himself expressed the wish that cognitive therapy as a school die out,^30^ the apt thought here being that what matters is not the treatment model but rather the particular treatment qualities which are individually worthy of study.

CBT treatments for depression often suffer high drop-out rates.^31^ One possible explanation for this is a lack of attention to transference. Sometimes this may be because negative transference undermines the therapeutic collaboration, although ideally CBT therapists are trained in recognising and managing this.^7^ At other times it may be because psychotherapeutic relationships that are merely collaborative, rather than offering experiential work in the transference, do not hit the therapeutic spot. Recently, however, there has been a resurgence in the general theory of,^8^ clinical practice and treatment manual for,^32^ and outcome studies supporting a transference-involving psychoanalytic approach to depression. With regard to outcome, outstanding results in helping patients with treatment-resistant depression which is not readily resolved by CBT have been obtained by particular practitioners of psychoanalytic psychotherapy^33^ and somewhat optimistic results have been obtained with similar patients in the multi-practitioner Tavistock Adult Depression Study.^34^

The present article has not been concerned to argue for a psychoanalytic approach to depression on the basis of empirical evidence. Instead, it noted that Beck's development of a cognitive approach to depression was predicated on his rejection of a psychoanalytic understanding of depression in particular, of the dynamic unconscious in general, and of psychoanalytic psychotherapeutic methods – and that his rationale for all this was flawed. What he developed in its stead does not stand or fall on this basis, and there are several benefits (especially clinical pragmatism and a strong research tradition) to the therapy he developed. But, given both the flawed rationale for rejecting a psychoanalytic approach which, rightly understood, possesses considerable clinical plausibility, and given the relatively poor results obtained by CBT for depression in much clinical practice, the time is surely right to revisit the psychoanalytic model.
